# Refining computer-assisted SEEG planning with spatial priors – A novel comparison of implantation strategies across adult and paediatric centres

**DOI:** 10.1016/j.neucli.2024.103038

**Published:** 2025-01-13

**Authors:** Debayan Dasgupta, Aswin Chari, Mehdi Khan, Friederike Moeller, Zubair Tahir, Andrew W McEvoy, Anna Miserocchi, John S Duncan, Rachel E. Sparks, Martin Tisdall

**Affiliations:** a Victor Horsley Department of Neurosurgery, National Hospital for Neurology and Neurosurgery, Queen Square, London, WC1N 3BG, UK; b Department of Clinical and Experimental Epilepsy, UCL Queen Square Institute of Neurology, University College London, London, WC1N 3BG, UK; c Developmental Neurosciences, UCL Great Ormond Street Institute of Child Health, London, WC1N 1EH, UK; d University College London Medical School, London, WC1E 6BT, UK; e Department of Neurosurgery, Great Ormond Street Hospital for Children, London, WC1N 3JH, UK; f Department of Neurophysiology, Great Ormond Street Hospital for Children, London, WC1N 3JH, UK; g School of Biomedical Engineering & Imaging Sciences, King’s College London, London, WC2R 2LS, UK

**Keywords:** Stereoelectroencephalography (SEEG), Computer-assisted planning, Spatial priors, Epilepsy surgery, Intracranial EEG, Surgical planning

## Abstract

**Objectives:**

Computer-assisted planning (CAP) allows faster SEEG planning and improves grey matter sampling, orthogonal drilling angles to the skull, reduces risk scores and minimises intracerebral electrode length. Incorporating prior SEEG trajectories enhances CAP planning, refining output with centre-specific practices. This study significantly expands on the previous work, compares priors libraries between two centres, and describes differences between SEEG in adults and children in these centres.

**Methods:**

98 adults and 61 children who underwent SEEG implantation as part of epilepsy surgery investigations were included. Priors libraries were created for each population, clustered by target regions and subdivided by cortical approaches. The libraries were coregistered and quantitatively and qualitatively compared.

**Results:**

The average number of implanted electrodes per patient was higher in paediatric patients than adults (13.6 vs 8.0). Paediatric implantations focused more on the insula than adult implantations (38.0 % vs 13.5 %), with similar proportions of electrodes implanted in the temporal and parietal lobes, and a higher proportion of adult electrodes in the frontal and orbitofrontal regions (40.6 % vs 24.0 %). Correspondence between the priors libraries was high. We present an example of a complex insular implantation planned with paediatric spatial priors and illustrate resultant SEEG recordings.

**Discussion:**

The use of centre-specific spatial priors allows the incorporation of surgeon-specific and unit-specific preferences into automated planning. We compare implantation styles between a paediatric and an adult centre, discussing similarities and differences. This tool allows centres to compare practice and represents an effective way to analyse implantation strategies that is agnostic to method of implantation.

## Introduction

Stereoelectroencephalography (SEEG) has rapidly grown in popularity worldwide as the most frequently used stage 2 presurgical investigation in the localisation of the putative seizure onset zone [6,22]. Precise planning of SEEG implantations is essential to maximise desired sampling of regions of interest, while simultaneously minimising the risk of symptomatic haemorrhage (2–3 %)[13,16,17].

We have previously demonstrated that computer-assisted planning (CAP) with the use of accurate vascular models, results in faster SEEG planning and improves grey matter sampling, orthogonal drilling angles to the skull, reduces risk scores (optimising trajectories away from intracerebral vasculature), and reduces the average intracerebral length of electrodes [18,24]. A further pilot study suggests that reference to prior SEEG trajectories through the creation of spatial prior trajectories enhances CAP [25], allowing for the plans created with CAP to be individualized for centre-specific preferences, while still providing all of the advantages of safety metrics and automated planning.

In this work, we significantly expand upon this technique to further refine CAP-SEEG. We develop two detailed spatial priors libraries, one adult (98 patients) and one paediatric (61 patients) from two experienced comprehensive epilepsy centres. In this study, we compare these two prior libraries quantitatively and qualitatively with the aim of exploring and describing similarities and differences between SEEG explorations in adults and children in these centres. We also provide an exemplar case of a complex insular implantation to illustrate the utility of CAP-SEEG, highlighting the benefits of using individualised prior libraries.

## Methods

### Patient inclusion

98 adult and 61 paediatric patients were included who underwent depth electrode implantation for SEEG as part of their presurgical investigations at The National Hospital for Neurology and Neurosurgery (adult), or Great Ormond Street Hospital for Children (paediatric), London, U.K between 2015 and 2020. Patients were eligible for inclusion if they were undergoing their first SEEG implantation and had no previous resections or large structural abnormalities that would affect the brain segmentation and parcellation.

All patients underwent a standardized expert multi-disciplinary clinical assessment by specialist epilepsy neurologists, neurosurgeons, neuropsychologists, neuropsychiatrists and neurophysiologists. Electrode implantation schemes including targets of electrophysiological interest and adjacent eloquent cortex, were created based on an estimation of the likely seizure onset zone synthesized from all preceding presurgical evaluations, including clinical history, semiology, scalp EEG/video telemetry, psychometric evaluations, structural and functional MRI, as well as PET and SPECT imaging when appropriate.

### Ethical approval

Ethical approval for this study was provided by the Health Research Authority: 12/LO/0377. In the adult population, all adult patients included in the study provided written consent to the use of the EpiNav™ planning software and for inclusion in research studies. In the paediatric population, the project was registered with the Great Ormond Street Hospital (GOSH) R&D Office (19BI26) and individual patient informed consent was waived as it involved retrospective use of routinely collected clinical data.

### Spatial priors libraries

Two extensive SEEG priors libraries were generated using 763 trajectories from 98 previous adult implantations and 829 trajectories from 61 previous paediatric implantations, defining 31 targets and 51 entry zones from consecutive implantations performed in 2015–2020.

Each of the implanted trajectories was reviewed and categorized into a target and entry region relevant to the classified spatial priors, and each entry or target spatial prior ellipsoid was modelled as a multivariate Gaussian, where the centre of the ellipsoid is the mean of the electrodes categorized into being, for example “anterior cingulate, superior approach” (meaning the target is the anterior cingulate gyrus, and the entry ellipsoid is those electrodes that approached this target through a superior approach, commonly the lateral superior frontal gyrus or the dorsal half of the middle frontal gyrus at our adult centre). The spread of the ellipsoid is then modelled as the standard deviation along the principal components.

Briefly, priors in each of the adult and paediatric cohorts were created as follows:

1. Each electrode was first clustered by its target region (e.g. amygdala, hippocampus)
2. Electrodes that were placed to target a lesion or patient specific abnormality were excluded as these are considered “patient specific” and therefore would not be helpful for guiding the implantation of a different patient with a different pathology.
3. Each group of electrodes were further subdivided into sub-target regions where there may be several distinct clinically relevant targets (e.g. anterior, middle and posterior hippocampus, anterior and posterior insula)
4. When appropriate, the priors were subdivided based on approach where there are distinct entry regions (e.g. lateral versus superior-oblique approaches to the anterior and posterior insula).
5. Once each group was identified, we:
  - excluded electrodes if they were clear outliers, defined as being outside of 1 standard deviation of the remaining electrodes in that group, and;
  - fitted an ellipsoid to be centred at the mean of the spread of the previously implanted electrodes in the group and having axis along the three principal directions of variance with each axis length being two standard deviations from the mean (i.e. capturing 95 % variance).
6. All electrodes and ellipsoids were reviewed by two consultant neurosurgeons and 2 senior fellows to verify that the grouping and exclusions were justified, and that the resulting ellipsoids were anatomically what would be expected when overlaid on the MNI-152 (ICBM 2009a Non-linear Asymmetric) group template brain ^15,16^ within the 3-dimensional rendering of the planning software.

**Fig. 1:**
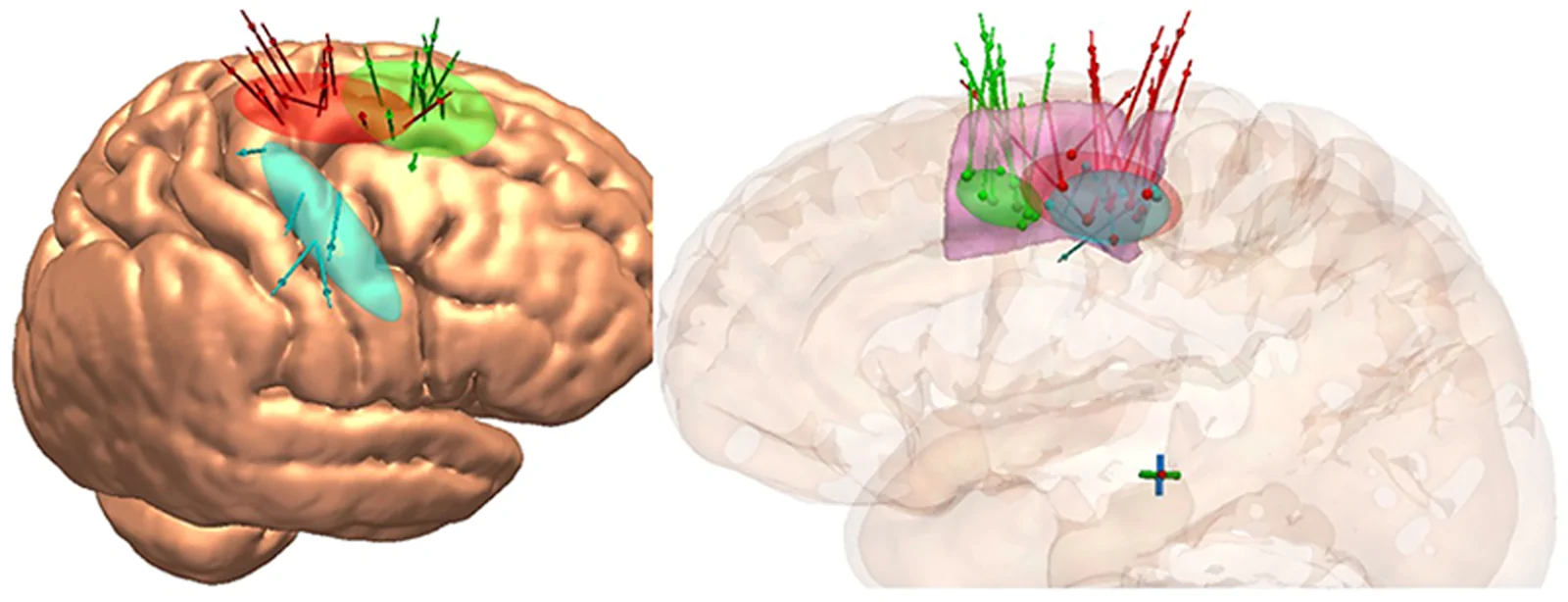
Left – example lateral (cyan), anterior (green), and posterior (red) entry point spatial priors with constituent previously implanted electrodes for the right supplementary motor cortex, demonstrated on a mesial to lateral (left to right) view of a 3-dimensional rendering of the cortical surface of the MNI-152 template [10]. Right – example target point spatial priors for the right supplementary motor cortex, demonstrated on a 3-dimensional model of the semi-opaque 3-dimensional reconstruction of the cortex of the MNI-152 template [10]. Cyan = right supplementary motor cortex lateral approach entry prior and electrodes, green = right supplementary motor cortex anterior approach entry prior and electrodes, red = right supplementary motor cortex posterior approach entry prior and electrodes, pink (in right panel alone) = right supplementary motor cortex parcellation derived from Geodesic Information Flow (GIF) version 3.0 [4].

Examples of the created entry and target priors for the supplementary motor cortex in Fig. 1, demonstrating both anterior and posterior approaches through the superior frontal gyrus and a lateral approach primarily through the middle frontal gyrus.

### Study design & statistical analysis

The resultant priors libraries and constituent electrodes were compared quantitively assessing for differences in proportions of electrodes implanted in each lobe using 2-tailed *z*-score tests for 2 population proportions, at a 0.05 significance level, with a Bonferroni correction to account for multiple comparisons. Statistical analyses were performed in IBM SPSS Statistics© v29 and RStudio.

We next performed a qualitative comparison: The target and entry prior ovoids for both populations were subsequently co-registered into the same atlas brain space and visually compared for correspondence of entry and target ovoids by 2 neurosurgeons. Correspondence was defined as direct spatial overlap as assessed on the 3-dimensional views in the planning software for each corresponding adult and paediatric prior ovoid, at both entry and target. This correspondence was then averaged across the entry and target ovoids for each prior, and categorized into excellent (>75 % spatial overlap of both the entry and target ovoids), good (50–75 % spatial overlap), and poor correspondence (<50 % overlap) to facilitate comparison and discussion around similarities and differences in approach and implantation.

Finally, we illustrate the value of refining CAP with priors libraries with a single case study from a complex insular paediatric SEEG implantation, comparing the CAP-SEEG plans produced when using paediatric and adult priors.

## Results

### Quantitative comparison: distribution of electrodes

Sixty-one paediatric and ninety-eight adult implantations were included, with eight hundred and twenty-nine and seven hundred and eighty-eight electrodes respectively. This equates to a mean 13.6 electrodes per paediatric SEEG implantation, and 8.04 electrodes per adult implantation. We interrogated the distribution of these electrodes by dividing them into the following lobes/regions: frontal, orbitofrontal, insula, temporal, parietal, and occipital. Results and statistical analyses are summarised in Table 1.

Nearly 40 % of all paediatric electrodes were implanted into the insula (vs 13.5 % in adults) (*p* < 10^−10^). In adults there was increased sampling of the frontal (*p*<.001), orbitofrontal, and occipital regions (both *p*<.00001). Sampling of the temporal and parietal lobes was similar in adults and children.

### Qualitative comparison: comparisons of priors ovoids

Throughout these results sections, we have divided the priors into regions for ease of comparison and provided comprehensive comparative tables illustrating each prior library for each region in Supplementary Tables 1–6. The figures below focus on those priors where there is minimal, or no correspondence between the adult and paediatric libraries.

### Frontal lobe

Supplementary Table 1 demonstrates the adult and paediatric priors in the frontal lobe, illustrating extent of correspondence, and names of priors used for planning implantations. As demonstrated in the table, there is excellent correspondence between the priors sampling the frontal lobe, with 5/11 with excellent correspondence (>75 %) in 3-dimensional space when overlaid on an atlas brain [10,11], and the remaining 6/11 with very good correspondence (>50 %).

### Orbitofrontal region

Supplementary Table 2 demonstrates the adult and paediatric priors in the orbitofrontal region, illustrating extent of correspondence, and names of priors used for planning implantations. As with the frontal lobe, there is a predominance of orbitofrontal sampling in the adult implantations when compared to paediatric SEEG implantations (13 % vs 5 %, *p*<.00001).

**Fig. 2:**
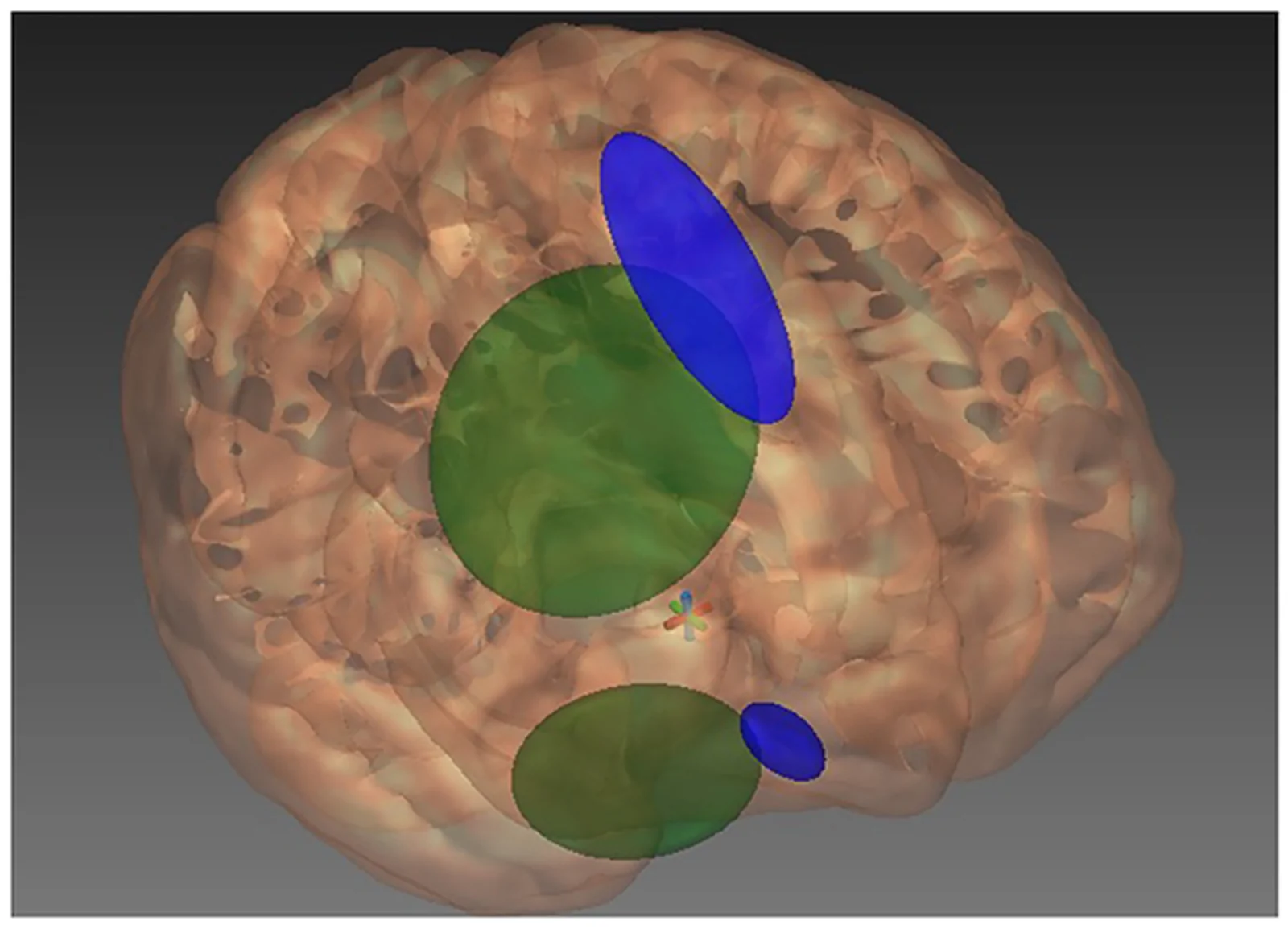
Illustrating the adult orbitofrontal cortex – superior approach prior ovoids (in green) that do not have a corresponding paediatric prior. The paediatric mesial frontal oblique prior ovoids are shown in blue as the closest corresponding paediatric prior. Demonstrated on a frontal oblique view of the semi-opaque 3-dimensional reconstruction of the cortex of the MNI-152 template [10].

There is excellent correspondence between the adult and paediatric prior ovoids, with 3/4 with excellent or very good correspondence (>50 %) in 3-dimensional space when overlaid on an atlas brain [10,11]. There is one adult prior without a paediatric equivalent in this region – the oblique/superior approach to the lateral orbitofrontal cortex, illustrated in Fig. 2.

### Insula

**Fig. 3:**
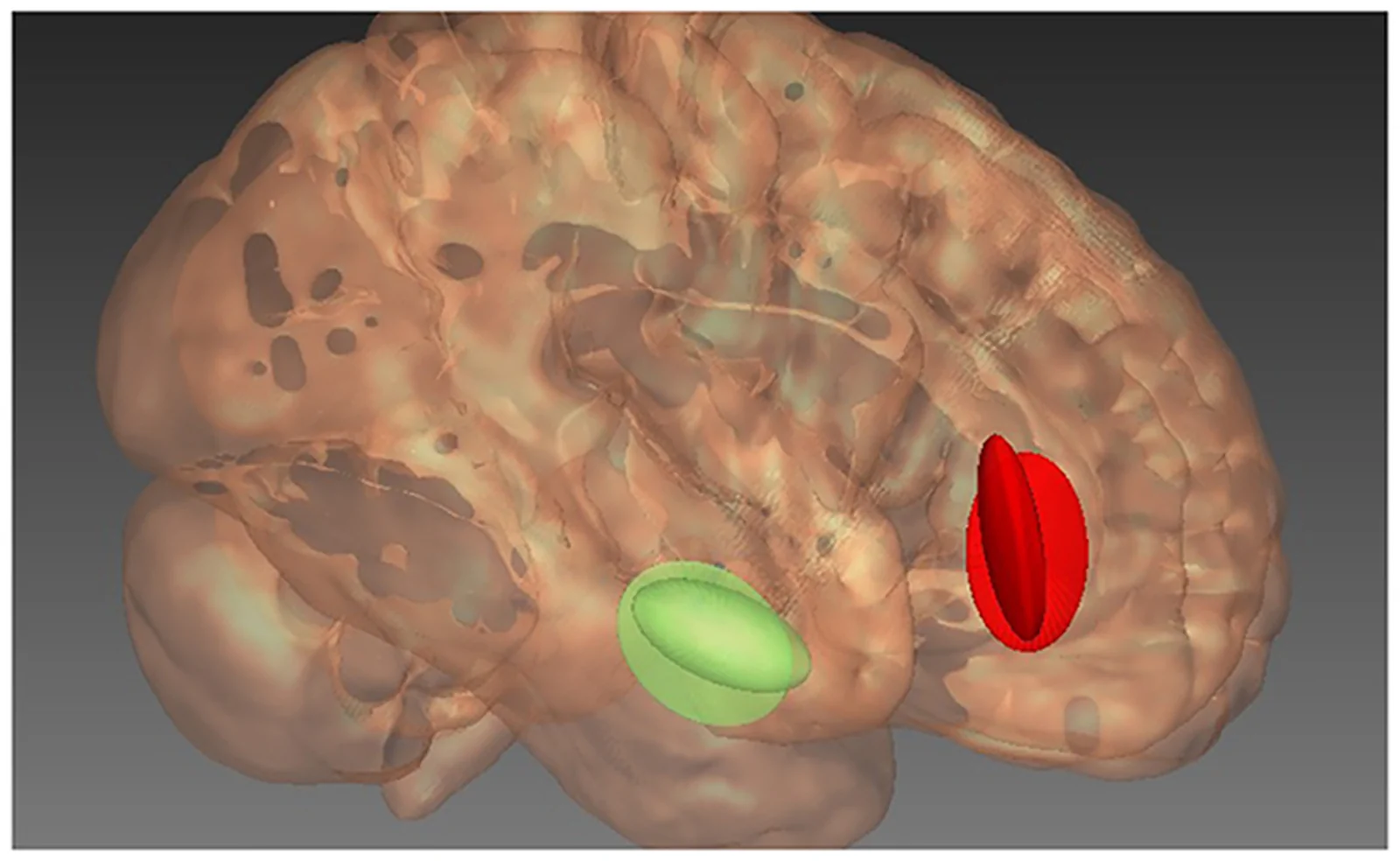
The temporal approach to the anterior insula paediatric prior entry and target ovoids are illustrated in light green, demonstrating lack of correspondence with the nearest equivalent adult prior illustrated in red (the pars triangularis adult prior). Demonstrated on a lateral view of the semi-opaque 3-dimensional reconstruction of the cortex of the MNI-152 template [10].

Supplementary Table 3 demonstrates the adult and paediatric priors in the insula, illustrating extent of correspondence, and names of priors used for planning implantations. The insula is where there is the biggest variation in practice between paediatric and adult SEEG implantations. There are more electrodes in paediatric (38.0 %) than adult (13.5 %) SEEG implantations (*p* < 10^−10^). There are also more electrodes per prior ovoid in the paediatric priors library than in the adults. In the paediatric series there is a lateral temporal approach to the anterior insula through the superior temporal gyrus that is not present in adult implantations, illustrated in Fig. 3. There remains good overall correspondence between the 2 prior libraries with 7/9 priors having excellent (>75 %) or very good (>50 %) correspondence.

### Temporal lobe

**Fig. 4:**
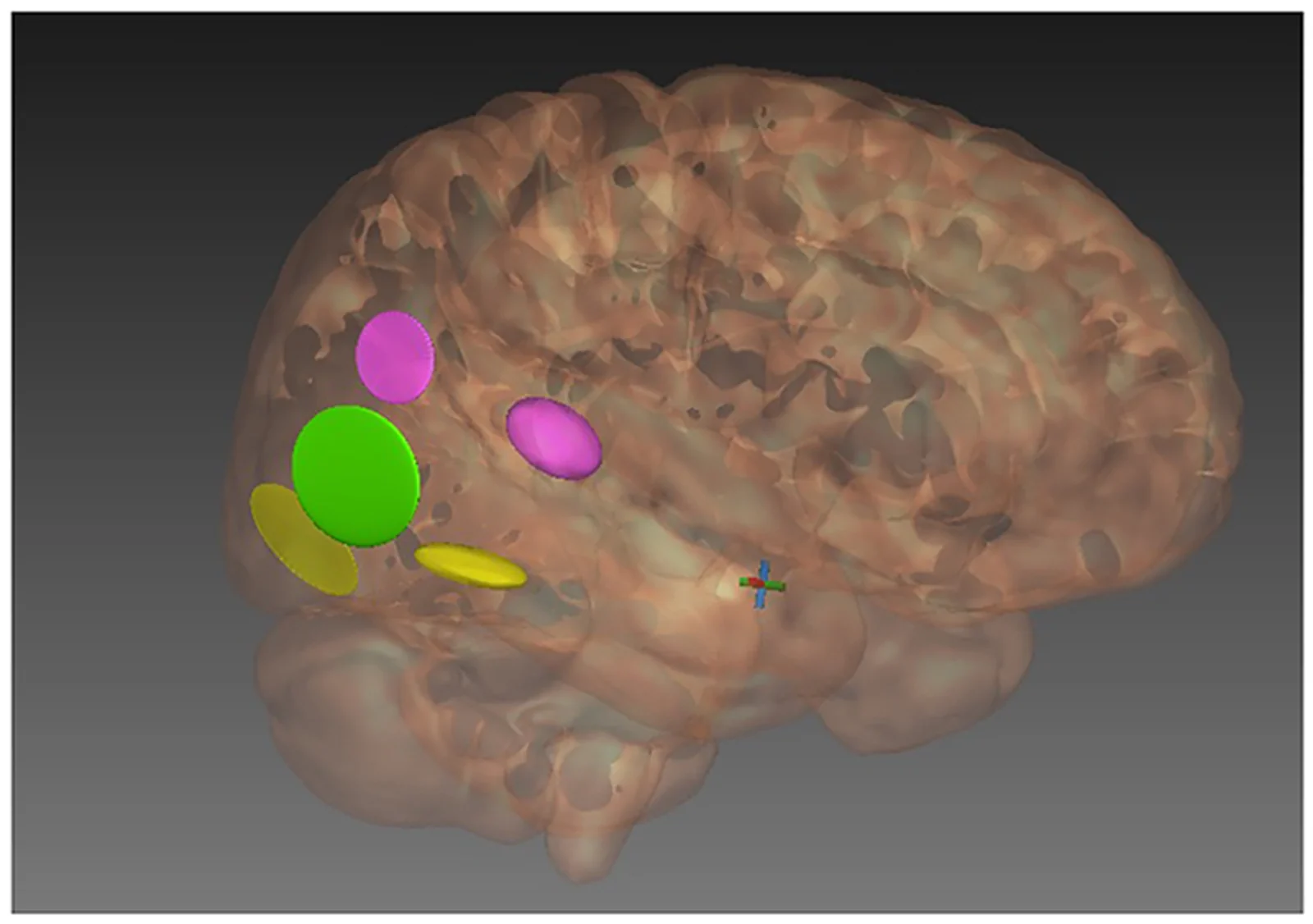
The posterior basal temporal (yellow) and Heschl’s gyrus (pink) paediatric prior ovoids illustrated on a lateral oblique view of the semi-opaque 3-dimensional reconstruction of the cortex of the MNI-152 template [10]. The entry ovoid of the adult posterior hippocampus prior is included (in light green) as the nearest adult equivalent.

The classical orthogonal trajectories targeting the hippocampus, amygdala, and temporal pole demonstrate excellent correspondence (Supplementary Table 4). There is a higher number of adult prior electrodes for the hippocampus and amygdala. In the paediatric series there were also prior ovoids targeting the posterior basal temporal lobe, and Heschl’s gyrus, that did not feature in the adult cohort, as illustrated in Fig. 4.

### Parietal lobe

**Fig. 5:**
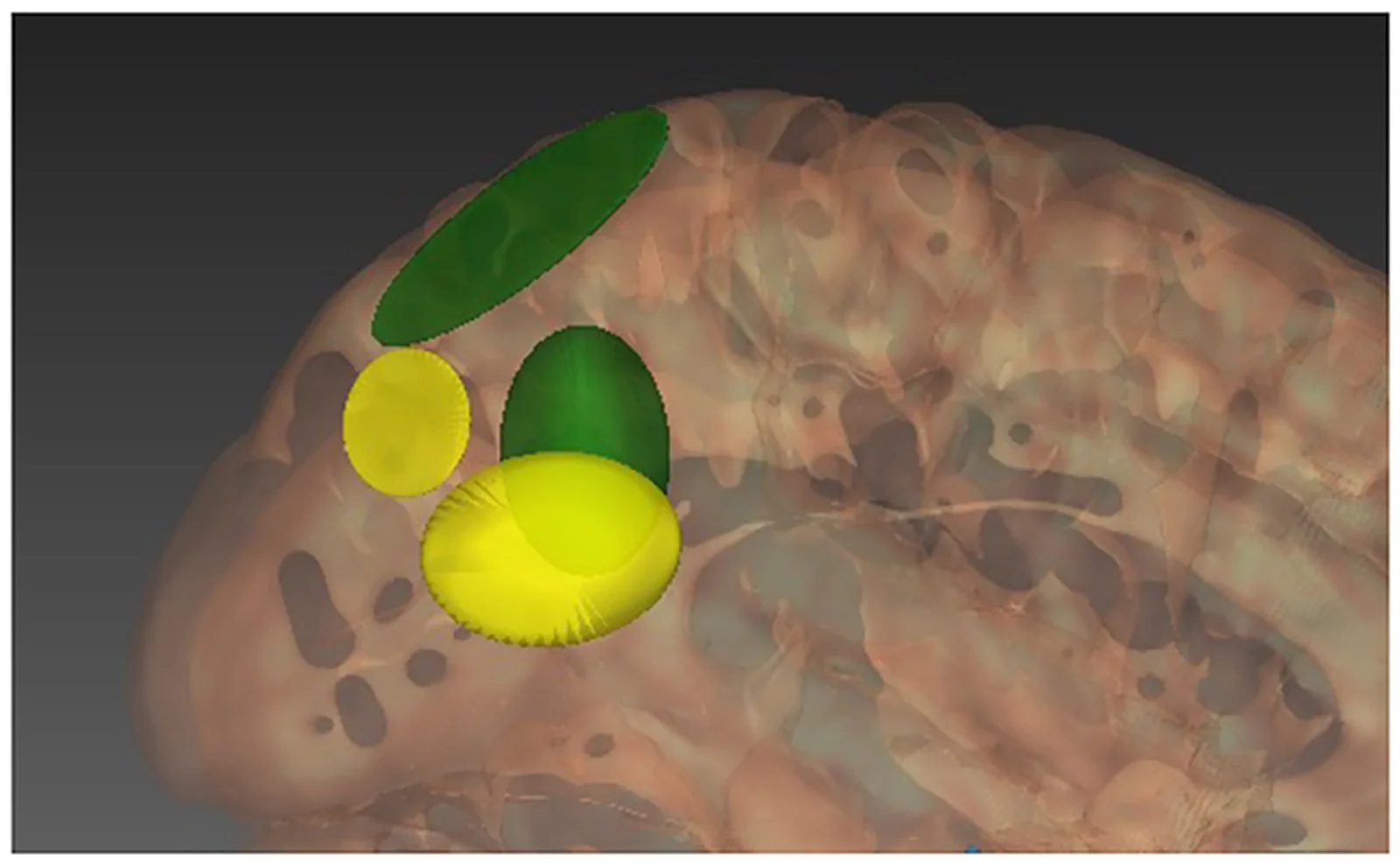
The paediatric temporo-occipito-parietal junction prior ovoids (yellow) illustrated on a lateral oblique view of the semi-opaque 3-dimensional reconstruction of the cortex of the MNI-152 template [10]. The adult precuneus, lateral approach entry and target ovoids (dark green) are included as the nearest adult equivalents, showing no entry correspondence, but some target correspondence.

**Fig. 6:**
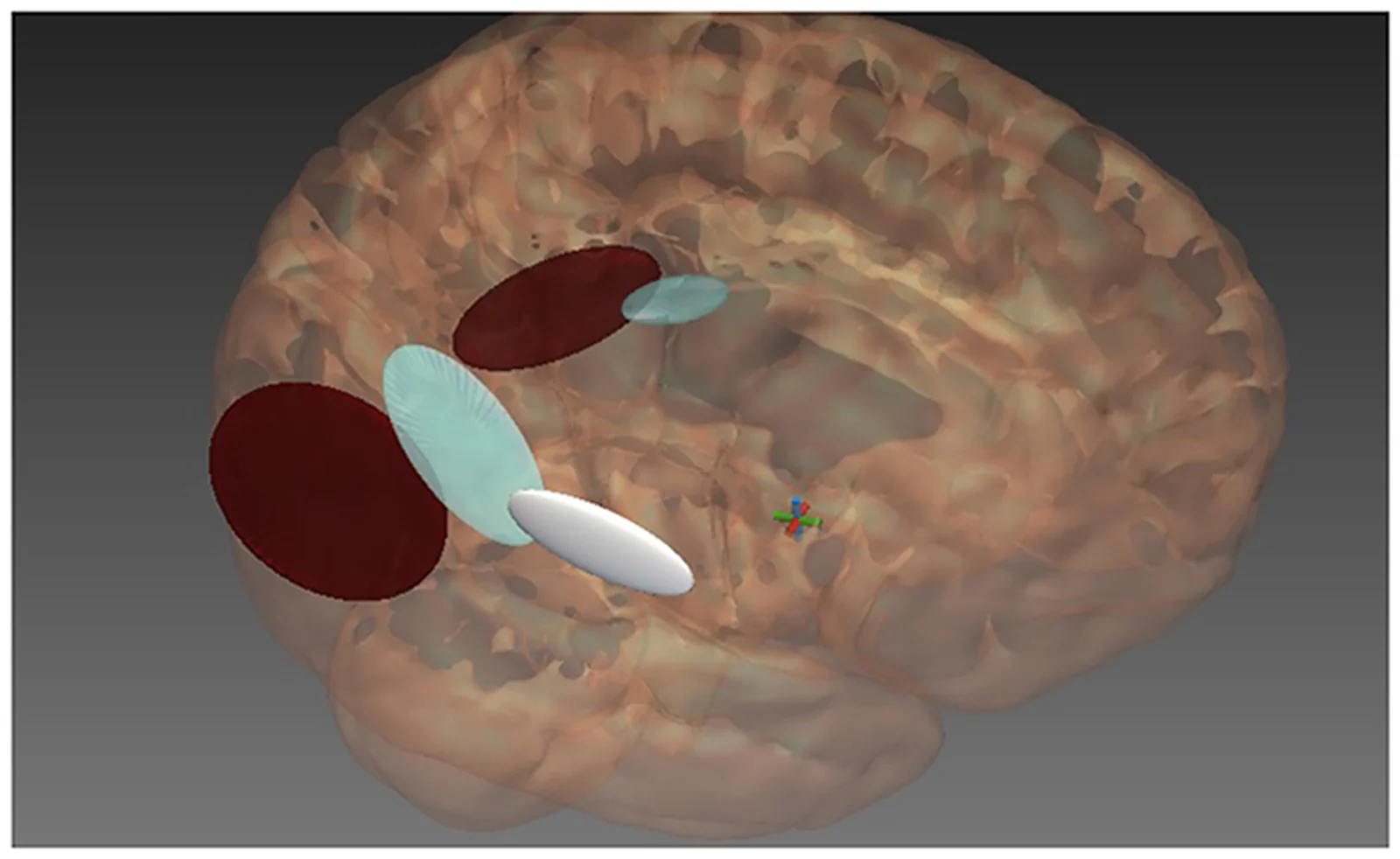
The paediatric lateral post-central gyrus to posterior cingulate prior ovoids (light blue) illustrated on a lateral oblique view of the semi-opaque 3-dimensional reconstruction of the cortex of the MNI-152 template [10]. The adult posterior cingulate, lateral approach (burgundy) entry and target prior ovoids, and the adult central operculum entry prior ovoid (white) are included as the nearest adult equivalents showing minimal spatial correspondence.

The spatial prior ovoids from both the adult and paediatric cohorts demonstrate distinct prior groupings with tight ovoids that correspond very well (8/10 > 50 % correspondence, Supplementary Table 5). There is a similar proportion of overall sampling in each cohort, but there are 2 prior ovoids in the paediatric implantations that are not represented in the adult cohort – the temporo-occipito-parietal junction prior (Fig. 5), and the lateral post-central gyrus to posterior cingulate prior, that is just posterior to the adult central operculum prior, and just anterior to the adult posterior cingulate, lateral approach prior (Fig. 6).

### Occipital lobe

**Fig. 7:**
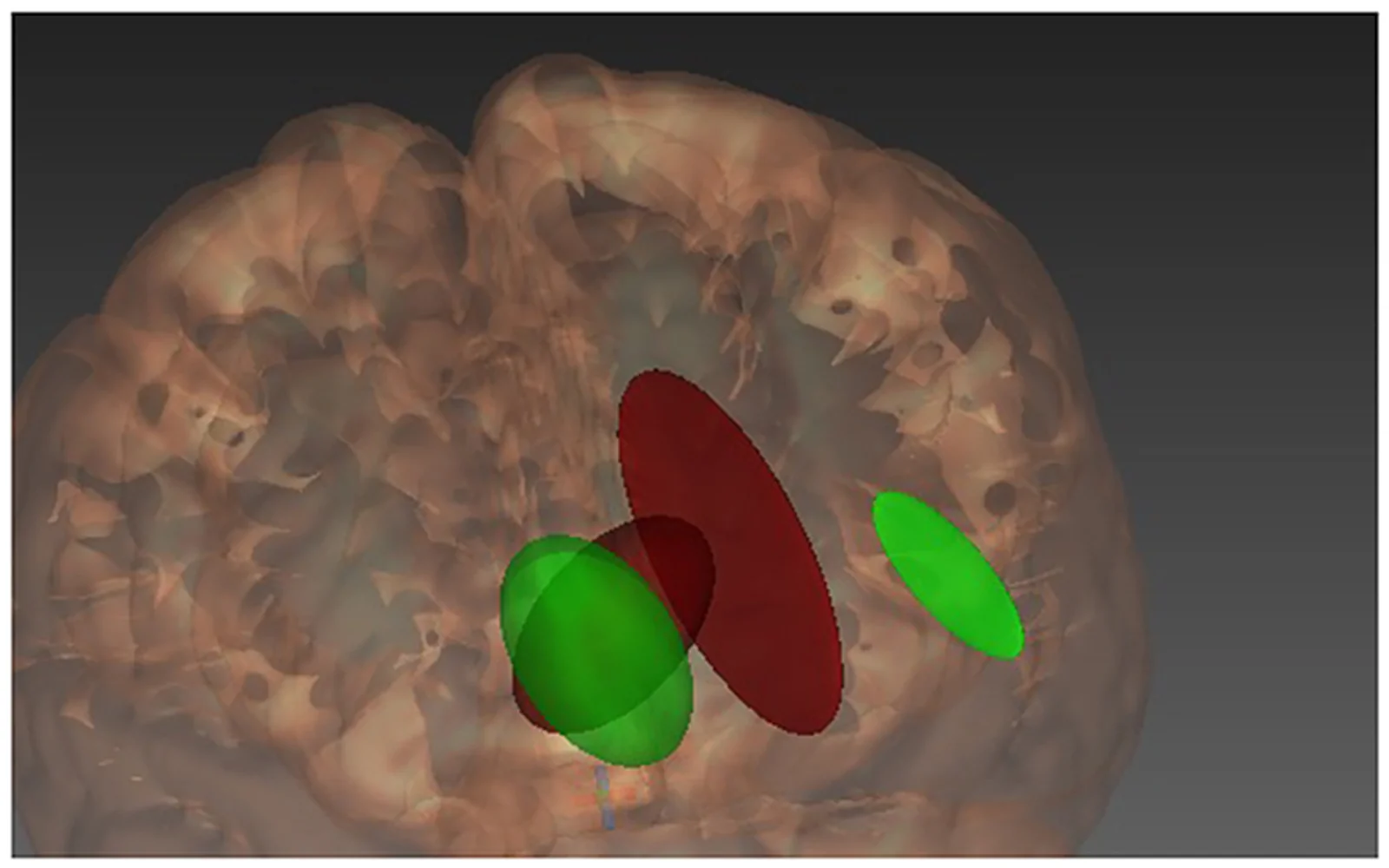
The adult posterior approach to the Calcarine cortex entry and target prior ovoids (burgundy) illustrated on a posterior oblique view of the semi-opaque 3-dimensional reconstruction of the cortex of the MNI-152 template [10]. The paediatric occipital infracalcarine (light green) entry and target prior ovoids are included demonstrating good target correspondence but no entry correspondence.

**Fig. 8:**
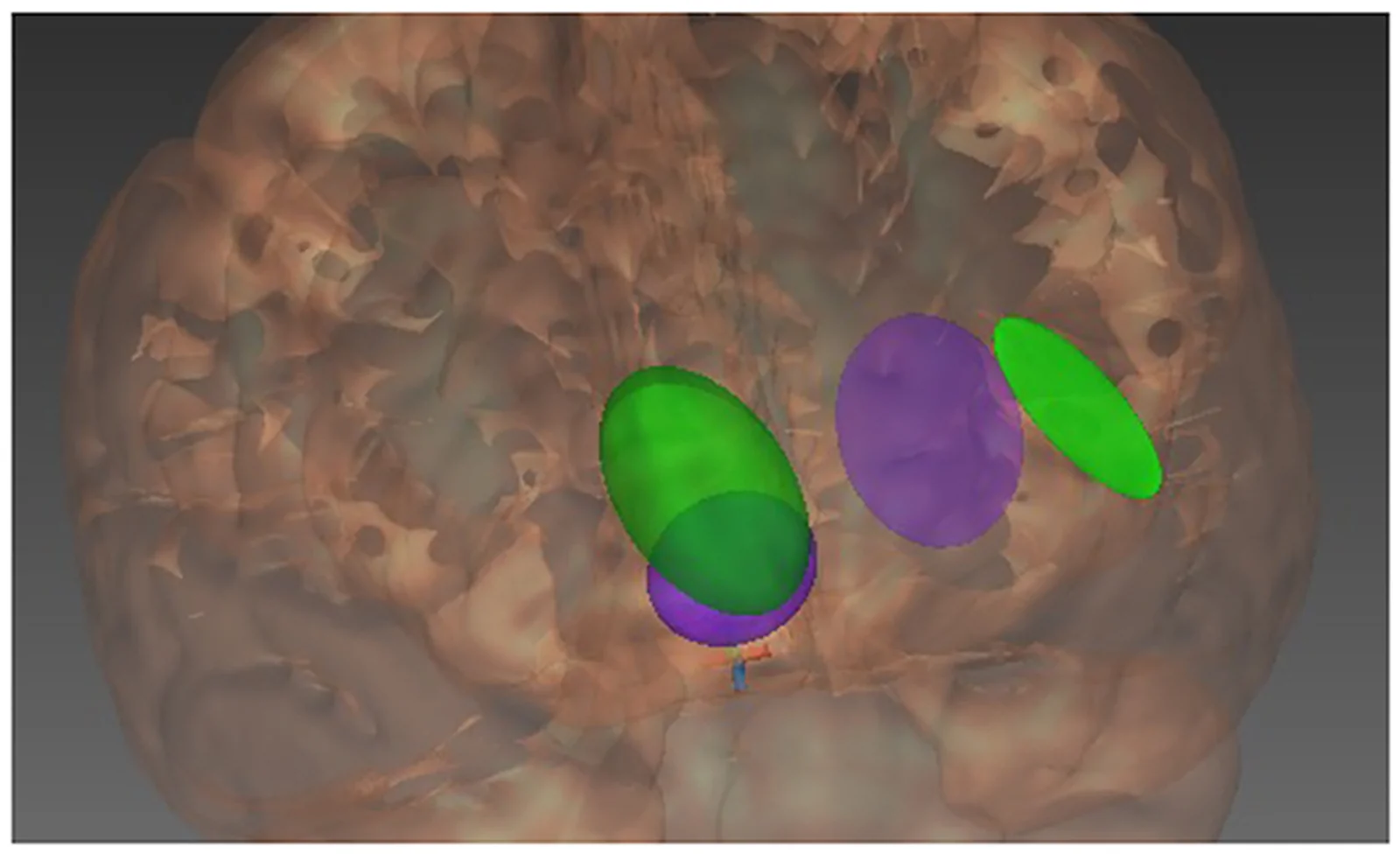
The adult posterior approach to the lingual gyrus entry and target pior ovoids (purple) illustrated on a posterior oblique view of the semi-opaque 3-dimensional reconstruction of the cortex of the MNI-152 template [10]. The paediatric occipital infracalcarine (light green) entry and target prior ovoids are included demonstrating good target correspondence but no entry correspondence.

The occipital lobe is sampled more often in adult than paediatric implantations (10 % vs 3 %, *p*<.000001, Supplementary Table 6). There are 2 adult priors that do not have equivalents in the paediatric priors library – the posterior approaches to both the calcarine cortex (Fig. 7), and the lingual gyrus (Fig. 8).

### Exemplar case – complex insular implantation

The below case study illustrates the utility of spatial priors in refining CAP-SEEG in a complex left insular paediatric implantation where the paediatric priors library was used to constrain and improve the CAP-SEEG output.

### Clinical summary

**Fig. 9:**
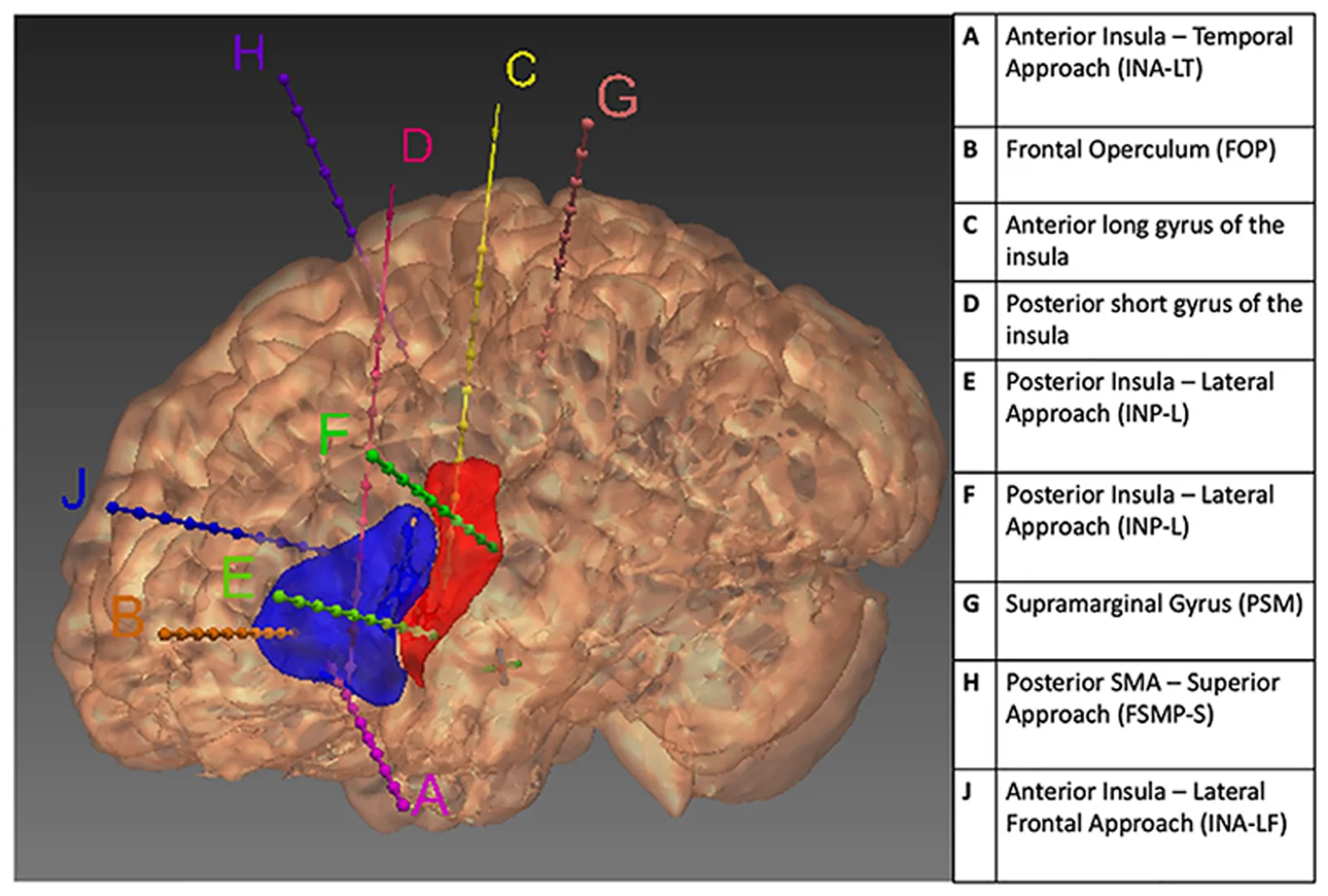
3-dimensional representation of desired left insula-focussed implantation, with table of electrodes (right) planned with the novel paediatric priors library next to the output electrodes of CAP-SEEG using the paediatric priors (codes for these priors in parentheses that match the summary Tables 2–7). Blue = left anterior insula parcellation, and Red = left posterior insula parcellation derived from Geodesic Information Flow (GIF) version 3.0 [4].

10-year-old child with nocturnal focal seizures, characterised by appearance of distress, with right arm posturing followed by guttural noises. Seizures occurred most nights, with bradycardia captured during telemetry recordings. Previous SEEG identified a seizure onset zone in the left posterior insula (along the anterior long gyrus), and the patient subsequently underwent thermocoagulation to that region with temporary seizure control. Subsequent laser ablation of the left anterior long gyrus of the posterior insula was performed and this led to 6 months of seizure freedom. However his habitual nocturnal seizures returned following this, at a frequency of 1–2/night. Repeat EEG demonstrated left centrosylvian ictal and interictal epileptic discharges. Positron-emission tomography demonstrated hypometabolism in the anterior long, and posterior short, gyri of the left insula, anterior to the location of the previous laser ablation. A re-exploration of the left insula with SEEG was performed, with a primary hypothesis of left anterior long/ posterior short gyri as the seizure onset zone. A dense and specific insula-focussed implantation was desired; with 5 orthogonal insular electrodes (3 through the temporal lobe and 2 through the frontal operculum), 2 oblique insular electrodes (specifically targeting the anterior long gyrus, just anterior to the area of previous laser ablation, and the posterior short gyrus), and 2 further electrodes in the supplementary motor cortex and the supramarginal gyrus. The desired implantation plan with paediatric priors-constrained CAP-SEEG is demonstrated in Fig. 9.

**Fig. 10:**
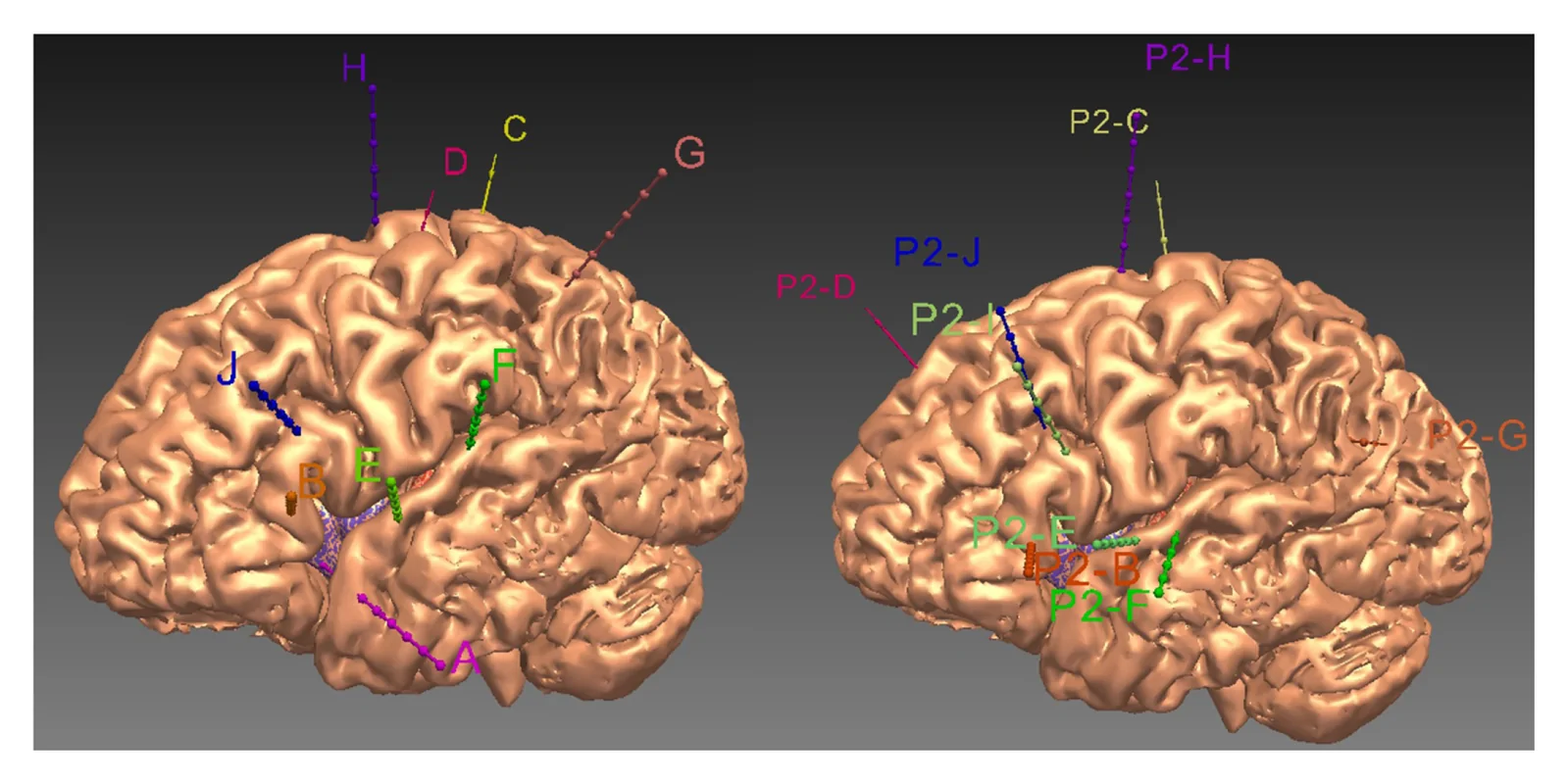
Left - 3-dimensional cortical model of the patient’s brain, with the paediatric electrodes that were planned with paediatric priors overlaid (electrodes labelled as in Fig. 2). Right – 3-dimensional cortical render of the patient’s brain, with the CAP-SEEG output with adult priors constraints applied (Electrodes labelled “P2-X”, with corresponding letters to Fig. 2).

We also retrospectively planned this implantation with the adult priors library on the same patient, and the results of this are illustrated alongside the paediatric implantation in Fig. 10:

As shown in Fig. 10, the adult priors library produced a viable implantation plan, but did not provide the flexibility to plan a second posterior oblique trajectory to the posterior short gyrus of the insula (it reverted to a more traditional anterior oblique trajectory, through the superior frontal gyrus). It also found 2 viable trajectories through the superior temporal gyrus, but did not find a third viable trajectory similar to the paediatric output. The adult priors CAP output found an additional approach to the posterior short gyri (labelled “P2-I” in Fig. 3), in a very similar location to electrode J in the paediatric plan, providing an alternative orthogonal option to sampling this area that was sampled obliquely in the paediatric implantation. This illustrates the flexibility of this approach, and also the differences in automated output of viable electrode trajectories that result from constraining the CAP with unit-specific prior trajectories.

**Fig. 11:**
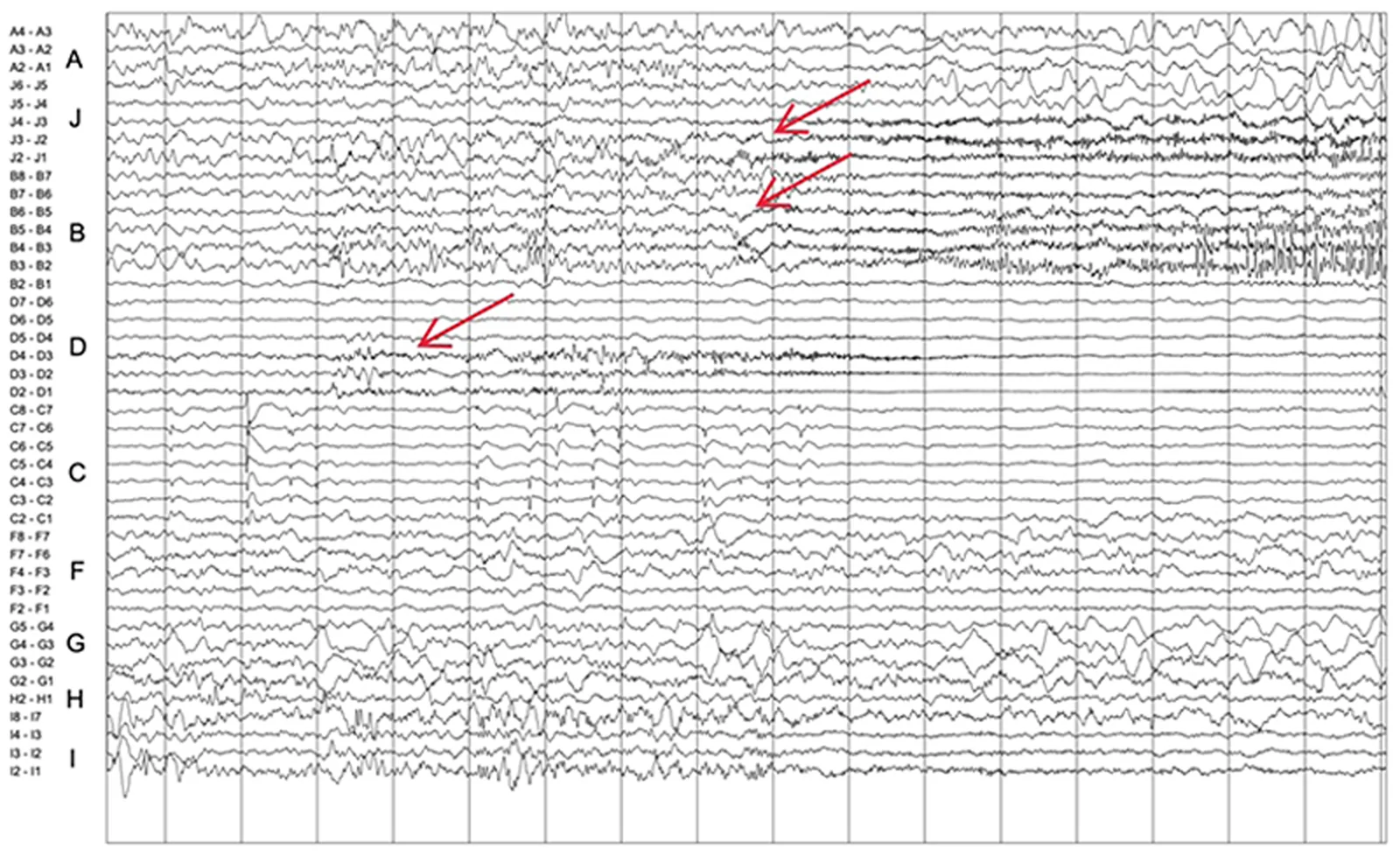
SEEG montage highlighting (with red arrows) fast activity first noted over electrode D (anterior long gyrus of the insula) with subsequent build-up of fast activity over B and J (posterior and mid short gyrus of the insula).

**Fig. 12:**
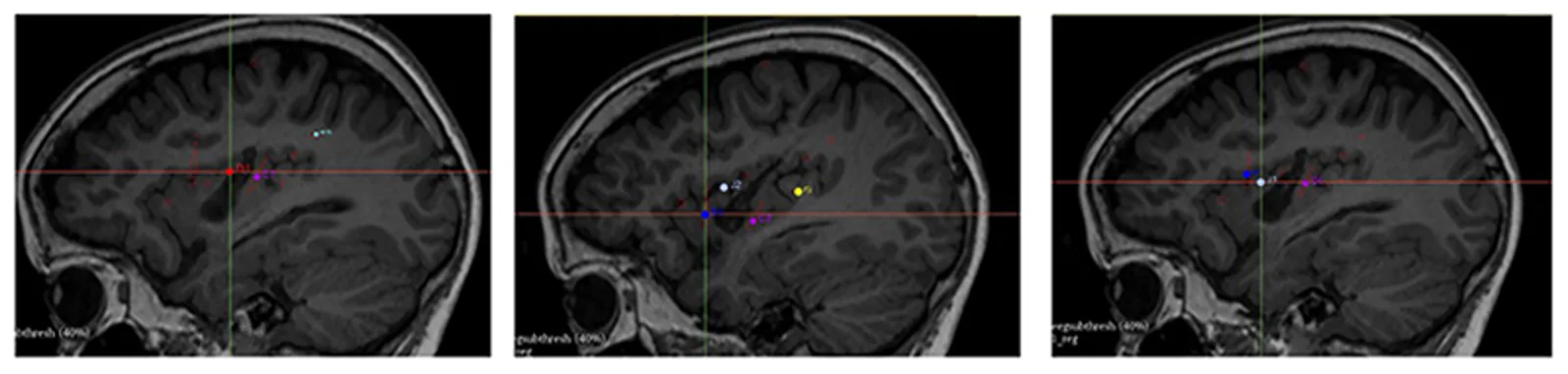
Sagittal T1-weighted volumetric MRI slices demonstrating the location of the 3 highlighted involved contacts on electrodes D (left, red), B (middle, dark blue) and J (right, light blue) shown in Fig. 3, all anterior to the area of laser ablation just posterior to the highlighted contact D1 (red). Crosshair highlights the location of the contact highlighted in Fig. 4 showing fast activity.

Figs. 11 and 12 demonstrate the fast activity commencing at electrode D (posterior oblique approach to the posterior short gyrus, just anterior to the previous area of laser ablation, that is visible as a hypointensity in the sagittal T1-weighted MRI in Fig. 5) and spreading quickly to electrodes B and J (middle and posterior short gyri of the insula respectively). Following this, the patient is currently awaiting a repeat laser ablation treatment to the identified region anterior to the previous ablation cavity.

## Discussion

This study demonstrates that the use of centre-specific spatial priors allows the incorporation of surgeon-specific and unit-specific preferences into automated planning for SEEG in both adults and children. This tool also allows centres to compare practice and represents an effective way to analyse implantation strategies that is agnostic to method of implantation.

This method allows structured analysis, comparison, and learning between epilepsy surgery centres’ approaches to SEEG implantation. This technique of creating spatial priors libraries from previous implantations at expert centres creates a systematic framework to review, compare and contrast the implantation styles of centres, and gives visual aids and frameworks to planning these implantations when using CAP-SEEG, that are easily adapted to patient-specific requirements. This represents a step forward in improving the flexibility and granularity of CAP-SEEG, and allows the automated planning to learn from previous implantations and preferences of a specific centre /surgeon, and from multiple centres and surgeons.

The results demonstrate that there are very high levels of similarity between the adult and paediatric priors libraries, with 31/39 comparative priors in each library having excellent or very good correspondence (>50 % spatial overlap in 3 dimensions across both entry and target ovoids), and 6 of the remaining 8 having at least 25 % spatial overlap at both entry and target points (demonstrated in detail in Supplementary Tables 1–6. This suggests the 2 different centres implant the same regions of the brain in a similar fashion. This also demonstrates how this methodology allows easy comparison between differing preferences between centres.

The differences in implantation styles observed in this study are likely primarily due to differences in preferences of the respective multidisciplinary teams in the centres involved in the study. We briefly speculate on the causes of some of these observed differences below, but acknowledge that the design of this study does not allow detailed analysis of the specific causes of observed differences. Differences in ethos and targeting styles is an area of much discourse among epilepsy surgery centres of excellence around the world, and the debate surrounding orthogonal and oblique trajectories, especially when targeting difficult to sample regions such as the insula is well-explored in the literature [2,7,8,12,21], particularly with the advent of robotic-assisted SEEG implantation [9,20]. This has been explored in detail in the literature elsewhere [6,23], but is worth noting that both the adult and paediatric priors in the insula, orbitofrontal, and frontal lobes in particular demonstrate extensive use of both orthogonal and oblique trajectories (Supplementary Tables 1–3). This is likely to be facilitated by the fact that both centres use robotic-assisted implantation techniques (the paediatric centre uses Neuromate® (Renishaw, Wotton-under-Edge, Gloucestershire, United Kingdom) with a frame-based implantation technique, and the adult centre uses Stealth AutoGuide® (Medtronic, Minneapolis, USA) with a frameless implantation technique), allowing for the stability and accuracy of implantation to allow for the increased drilling angles to bone often encountered with oblique trajectories. This reinforces the conclusions of other groups that anterior and posterior oblique trajectories represent a safe and viable method of increasing SEEG sampling of the insula [3,14,20].

This study also demonstrates the utility of Priors in planning a complex insular implantation, combining orthogonal and oblique trajectories in a case that requires extensive insular sampling – CAP + Priors allows comprehensive insulo-opercular coverage with up to 7 electrodes (5 orthogonal and 2 oblique, Fig. 9), facilitating detailed mapping of all the insular gyri to delineate the location and borders of a putative insular epileptogenic zone.

It is also interesting to note the additional prior in the paediatric library that is not in the adult cohort – an orthogonal approach to the anterior insula through the superior temporal gyrus (STG) which adds to the insular coverage possible, while doing so through the anterior-mid STG that is generally otherwise not used to sample mesial temporal structures: The majority of electrodes targeting the amygdala and hippocampus enter through the middle temporal gyrus in both adult and paediatric priors, again demonstrating good correspondence between the implantation styles of the 2 centres (Supplementary Table 4).

A difference in multidisciplinary team ethos may also explain the lack of a lateral orbitofrontal cortex electrode prior in the paediatric library, as this cortex is often sampled *en passant* by the mesial orbito-frontal electrodes from a lateral approach (in red in Supplementary Table 2). In the adult cohort, while there is a similar lateral to medial orthogonal electrode, often there is a request for a specific targeting of the lateral orbitofrontal cortex when the semiology suggests this may be involved, and so a second oblique electrode is placed in this region, though this is often difficult to plan, and leads to high drilling angles to bone to avoid entering too low on the subject’s forehead.

Another observed difference in the priors libraries created is the predominance of priors (and therefore electrodes) that are targeted at the entry point (and the cortex that is entered for sampling) in the paediatric cohort, whereas the adult centre tends to plan more consistently based on deep cortical targets – this is reflected in the resulting priors libraries, where while there is overall a similar amount of lateral neocortical coverage, the naming of comparable electrode groupings is different from the different centres. This is reflected in the existence of paediatric priors that target Heschl’s gyrus (Fig. 4), the lateral post-central gyrus (S2, Fig. 6), temporo-occipito-parietal (TOP) junction (Fig. 5), and Wernicke’s area (Supplementary Table 5). All these priors are targeted at sampling lateral neocortex on entry, but match up with adult priors named by the deep cortex they target (for example the Wernicke’s paediatric prior corresponds well to the posterior cingulate cortex lateral approach adult prior ovoids) However, there remain a minority of priors where the correspondence in spatial overlap is lower - Heschl’s gyrus, S2, and the TOP junction (where, for example there is some overlap between the precuneus lateral approach adult entry prior and the paediatric TOP junction entry prior).

Another difference in ethos of the centres is the avoidance of posterior approaches to the occipital lobe in paediatric implantations, as the paediatric centre feels children have difficulty sleeping with these electrodes - this may account for the lack of correspondence for both the posterior approach adult priors to the calcarine cortex and the lingual gyrus illustrated in Figs. 7 and 8. This is also reflected in the lower percentage of paediatric occipital electrodes (10 % in adults vs 2.9 % in the paediatric cohort, *p*<.00001). This problem can also be mitigated with the use of large, padded dressings around the posterior electrodes.

Differences in anatomy are more difficult to generalise across the paediatric and adult cohorts, however, for example the existence of a separate posterior temporal basal electrode prior in the paediatric library, in addition to both cohorts having a temporo-occipital junction (TOJ) prior, suggests the impact of more prominent mastoid sinuses in adults making trajectories that enter through the inferior temporal gyrus much more technically challenging, likely increasing drilling angle to bone.

Lesional cases, and a potentially higher proportion of these in the paediatric population [1,5,15,19] highlights a limitation of this technique of creating spatial prior ovoids to guide analysis of implantation preferences and SEEG planning, as lesional electrodes are removed from the priors libraries at the stage of grouping, as these are not generalisable from one subject to the next. While this is a limitation of the technique, we have found across both centres that this has not hampered our clinical standard computer-assisted planning for robotic-assisted SEEG implantations, as the use of priors has improved the granularity of our SEEG-CAP planning and sped this up, leading to a trend of less manual adjustments from the surgeon being required following the CAP-generated output. In addition, in lesional cases, the non-lesional electrodes are planned with CAP with priors, and subsequently the lesional electrodes are planned around this framework manually, speeding up the planning of the implantation as a whole, and giving the surgeon a better idea of the 3-dimensional sampling achieved.

Further work on implementing and validating the clinical use of these priors libraries in our CAP-SEEG implantations is ongoing at both the adult and paediatric centres involved. This systematic approach to collating centre-specific implantation styles provides an ideal framework to collate and develop significantly improved automated and computer-assisted planning tools that incorporate and assimilate expert input from multiple sites around the world into the automated output for streamlined, automated SEEG planning that is highly adaptable to surgeon and centre-specific preferences. This may be particularly useful in aiding less experienced centres, and those looking to set up a new SEEG services as part of a comprehensive epilepsy surgery unit, to learn from the experience and prior trajectories of more experienced centres. This technique allows a structured and objective way to adapt and analyse the CAP-SEEG output to maximise safety and grey matter sampling based on the practise of experienced centres.

## Conclusion

We have demonstrated that the use of centre-specific spatial priors allows the incorporation of surgeon-specific and centre-specific preferences into automated SEEG planning. This technique also creates a framework with which epilepsy centres around the world can discuss and learn from each other’s implantation styles and preferences. We compare in detail implantation styles between paediatric and adult expert centres, discussing similarities and potential sources of differences in ethos, nomenclature, and anatomy between the subject groups. This represents an exciting way to analyse implantation strategies that is agnostic to method of implantation, whether frame-based or frameless, robot-assisted or manual. Further work building on this technique to standardise and collate implantation styles could lead to significantly improved SEEG planning based on expert experience from centres around the world.

## Supplementary Material

Supplementary material associated with this article can be found, in the online version, at doi:10.1016/j.neucli.2024.103038.

Supplementary Material

## Figures and Tables

**Table 1:** Distribution of electrodes in adult and paediatric prior libraries.

Lobe	Paediatric	Percentage of total (3s. f.)	Adult	Percentage of total (3s. f.)	Bonferroni Corrected *p*-Value
**Frontal**	157	18.9	213	27.0	**< 0.001**
**Orbitofrontal**	42	5.07	107	13.6	**< 0.00001**
**Insula**	315	38.0	106	13.5	**< 0.000001**
**Temporal**	158	19.1	182	23.1	.279
**Parietal**	133	16.0	101	12.8	.392
**Occipital**	24	2.90	79	10.0	**< 0.00001**
**Total electrodes**	829		788		

Distribution of electrodes in adult and paediatric prior libraries. Yellow indicates significantly higher percentage of electrodes per SEEG implantation, bold indicates statistical significance in 2-tailed *z*-score tests for 2 population proportions, at a 0.05 significance level, with a Bonferroni correction.
