# Supplementary material for: Refining computer-assisted SEEG planning with spatial priors – A novel comparison of implantation strategies across adult and paediatric centres
Source: Neurophysiol Clin. Author manuscript; Available in PMC 2026 Sep 11. (PMC7619480; doi:10.1016/j.neucli.2024.103038)
Supplement: Supplementary file 1 [file EMS217848-supplement-Supplementary_Material.docx]

## Supplementary Material

For all supplementary tables the prior labels are the names used by each of the adult and paediatric centres when planning SEEG. The colours in each row are a base colour, and in general a lighter shade of that colour for visual comparison of the paediatric prior ovoids (e.g. light blue) to the adult prior ovoids (darker shade, e.g. dark blue). Where there is a combination of priors from one library that corresponds to the other cohort’s library of spatial priors, an arbitrary other colour is used to visually summarise this.

Supplementary Table 1: Paediatric and adult prior entry and target ovoids, number of constituent electrodes per prior, and level of correspondence (>75% is excellent, >50% very good, <50% poor) for the frontal lobe. Where there are significantly higher numbers of electrodes contributing to a prior in one population than the other, these are highlighted in blue.

Supplementary Table 2: Paediatric and adult prior entry and target ovoids, number of constituent electrodes per prior, and level of correspondence (>75% is excellent, >50% good, <50% poor) for the orbitofrontal region. Where there are significantly higher numbers of electrodes contributing to a prior in one population than the other, these are highlighted in blue. Where there is a prior that is not accounted for (or has poor correspondence) in the other cohort (paediatric or adult), this row is highlighted in green.

Supplementary Table 3: Paediatric and adult prior entry and target ovoids, number of constituent electrodes per prior, and level of correspondence (>75% is excellent, >50% good, <50% poor) for the insula. Where there are significantly higher numbers of electrodes contributing to a prior in one population than the other, these are highlighted in blue. Where there is a prior that is not accounted for (or has poor correspondence) in the other cohort (paediatric or adult), this row is highlighted in green.

Supplementary Table 4: Paediatric and adult prior entry and target ovoids, number of constituent electrodes per prior, and level of correspondence (>75% is excellent, >50% good, <50% poor) for the temporal lobe. Where there are significantly higher numbers of electrodes contributing to a prior in one population than the other, these are highlighted in blue. Where there is a prior that is not accounted for (or has poor correspondence) in the other cohort (paediatric or adult), this row is highlighted in green.

Supplementary Table 5: Paediatric and adult prior entry and target ovoids, number of constituent electrodes per prior, and level of correspondence (>75% is excellent, >50% good, <50% poor) for the parietal lobe. Where there are significantly higher numbers of electrodes contributing to a prior in one population than the other, these are highlighted in blue. Where there is a prior that is not accounted for (or has poor correspondence) in the other cohort (paediatric or adult), this row is highlighted in green.

Supplementary Table 6: Paediatric and adult prior entry and target ovoids, number of constituent electrodes per prior, and level of correspondence (>75% is excellent, >50% good, <50% poor) for the occipital lobe. Where there are significantly higher numbers of electrodes contributing to a prior in one population than the other, these are highlighted in blue. Where there is a prior that is not accounted for (or has poor correspondence) in the other cohort (paediatric or adult), this row is highlighted in green.
